# 4-Bromo-2-[5-methyl-2-(morpholin-4-yl)-1,3-thia­zol-4-yl]phenol

**DOI:** 10.1107/S1600536813017510

**Published:** 2013-06-29

**Authors:** Lucian G. Bahrin, Cristian G. Hrib, Lucian M. Birsa

**Affiliations:** a Department of Chemistry, "Al. I. Cuza" University Iasi, 11 Carol I Bvd, Iasi 700506, Romania; b Chemisches Institut der Otto-von-Guericke-Universität, Universitätsplatz 2, D-39116 Magdeburg, Germany

## Abstract

In the title compound, C_14_H_15_BrN_2_O_2_S, synthesized by the reaction of the corresponding phenacyl thio­cyanate with morpholine, the dihedral angle between the 1,3-thia­zole ring and the phenolic substituent ring is 23.46 (10)° as a result of the steric influence of the *ortho*-methyl group on the thia­zole ring. A strong intra­molecular phenolic O—H⋯N hydrogen bond is present in the mol­ecule. In the crystal, a weak C—H⋯O_phenol_ hydrogen bond gives rise to chains lying parallel to [20-1]. A short inter­molecular Br⋯O_morpholine_ inter­action is also present [3.1338 (19) Å].

## Related literature

For details of the synthesis, see: Seliger *et al.* (1997 ▶). For a recent review on thia­zoles, see: Zagade & Senthilkumar (2011 ▶). For the pharmacological activity and applications of thia­zole derivatives, see: Ghaemmaghami *et al.* (2010 ▶); Coco & Onnis (1993 ▶); Gewald *et al.* (1994 ▶); Tanaka *et al.* (1994 ▶); Zimmermann *et al.* (1990 ▶).
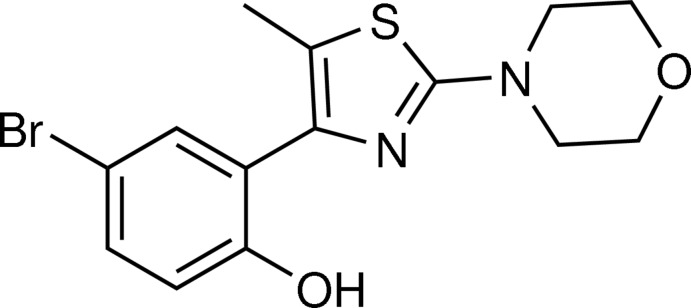


## Experimental

### 

#### Crystal data

C_14_H_15_BrN_2_O_2_S
*M* *_r_* = 355.25
Monoclinic, 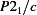

*a* = 12.026 (2) Å
*b* = 8.3448 (17) Å
*c* = 14.279 (3) Å
β = 91.98 (3)°
*V* = 1432.1 (5) Å^3^
*Z* = 4
Mo *K*α radiation
μ = 3.02 mm^−1^
*T* = 153 K
0.60 × 0.50 × 0.50 mm

#### Data collection

Stoe IPDS 2T area-detector diffractometer
Absorption correction: for a sphere [modification of the interpolation procedure of Dwiggins (1975 ▶)] *T*_min_ = 0.090, *T*_max_ = 0.117
9876 measured reflections
3848 independent reflections
3258 reflections with *I* > 2σ(*I*)
*R*_int_ = 0.049

#### Refinement

*R*[*F* ^2^ > 2σ(*F* ^2^)] = 0.038
*wR*(*F* ^2^) = 0.088
*S* = 1.11
3848 reflections
186 parameters
H atoms treated by a mixture of independent and constrained refinement
Δρ_max_ = 0.41 e Å^−3^
Δρ_min_ = −0.83 e Å^−3^

### 

Data collection: *X-AREA* (Stoe & Cie, 2002 ▶); cell refinement: *X-AREA*; data reduction: *X-RED* (Stoe & Cie, 2002 ▶); program(s) used to solve structure: *SHELXS97* (Sheldrick, 2008 ▶); program(s) used to refine structure: *SHELXL97* (Sheldrick, 2008 ▶); molecular graphics: *XP* in *SHELXTL* (Sheldrick, 2008 ▶); software used to prepare material for publication: *SHELXL97*.

## Supplementary Material

Crystal structure: contains datablock(s) I, global. DOI: 10.1107/S1600536813017510/zs2266sup1.cif

Structure factors: contains datablock(s) I. DOI: 10.1107/S1600536813017510/zs2266Isup2.hkl

Supplementary material file. DOI: 10.1107/S1600536813017510/zs2266Isup3.cml

Additional supplementary materials:  crystallographic information; 3D view; checkCIF report

## Figures and Tables

**Table 1:** Hydrogen-bond geometry (Å, °)

*D*—H⋯*A*	*D*—H	H⋯*A*	*D*⋯*A*	*D*—H⋯*A*
O1—H1⋯N1	0.88 (4)	1.79 (3)	2.603 (2)	154 (3)
C9—H9*C*⋯O1^i^	0.98	2.47	3.357 (3)	150

Symmetry code: (i) 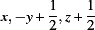.

**Fig. 1.**
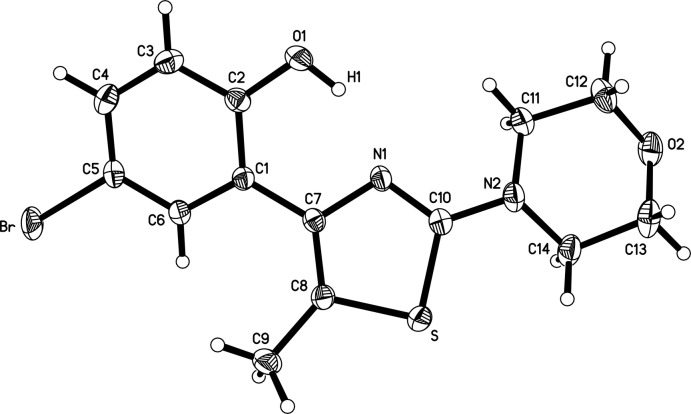
Molecular conformation and atom numbering scheme for the title compound, showing thermal ellipsoids drawn at the 50% probability level.

## supplementary crystallographic information

### Comment

Many derivatives of thiazole exhibit pharmacological activities (Coco & Onnis, 1993; Zagade & Senthilkumar, 2011)) and some of them are used as chemotherapeutic agents with anti-inflammatory, analgesic and antipyretic activities (Tanaka *et al.*, 1994). Recently, 2-aminothiazoles were described as a new class of small molecules with antiprion activity in prion-infected neuroblastoma cell lines (Ghaemmaghami *et al.*, 2010). On the other hand, thiocyanates play an important role in the chemistry of organosulfur compounds (Gewald *et al.*, 1994). Among these alkyl 2-thiocyanato phenylketones there are versatile precursors for various substituted thiazoles (Zimmermann *et al.*, 1990). The title compound, C_14_H_15_BrN_2_O_2_S, has been synthesized by the reaction of 1-(5-bromo-2-hydroxyphenyl)-2-thiocyanatopropan-1-one with morpholine and the structure is reported herein. In this compound (Fig. 1), although an intramolecular hydrogen bond is present between the phenolic O1—H group and N1 of the thiazole ring (Table 1), the benzene and thiazole rings are not coplanar, with a dihedral angle of 23.46 (10) ° between them. The steric influence of the methyl substituent group in the 5-position of the thiazole ring is considered to be responsible for this deviation. In the crystal, only a weak intermolecular C9—H···O1^i^ (phenol) hydrogen-bonding association is present, giving a one-dimensional chain lying parallel to [2 0 -1], while a short Br···O2^ii^ (morpholine) interaction [3.1338 (19) Å] is also found [for symmetry code (ii): *x* -1, -*y* + 1/2, *z* +1/2].

### Experimental

A mixture of 1-(5-bromo-2-hydroxyphenyl)-2-thiocyanatopropan-1-one (0.3 mmol) and morpholine (0.45 mmol) in 50 ml of methanol was heated under reflux for 20 min (Seliger *et al.*, 1997). After 24 h the reaction mixture was poured into water and the precipitate filtered off and dried. Recrystallization from methanol gave the pure product as colorless crystals, m.p. 415 K.

### Refinement

The C-bound H-atoms were included at calculated positions and treated using a riding model, with aromatic C—H = 0.95 Å and methylene C—H = 0.99 Å, and with *U*_iso_(H) = 1.2*U*_eq_(C), or with methyl C—H = 0.98 Å, with *U*_iso_(H) = 1.5*U*_eq_(C). The phenolic H-atom (H1) was freely refined.

### Crystal data

**Table:**

C_14_H_15_BrN_2_O_2_S	*F*(000) = 720
*M**_r_* = 355.25	*D*_x_ = 1.648 Mg m^−^^3^
Monoclinic, *P*2_1_/*c*	Melting point: 415 K
Hall symbol: -P 2ybc	Mo *K*α radiation, λ = 0.71073 Å
*a* = 12.026 (2) Å	Cell parameters from 13229 reflections
*b* = 8.3448 (17) Å	θ = 2.3–29.5°
*c* = 14.279 (3) Å	µ = 3.02 mm^−^^1^
β = 91.98 (3)°	*T* = 153 K
*V* = 1432.1 (5) Å^3^	Prism, colourless
*Z* = 4	0.60 × 0.50 × 0.50 mm

### Data collection

**Table:**

Stoe IPDS 2T area-detector diffractometer	3848 independent reflections
Radiation source: fine-focus sealed tube	3258 reflections with *I* > 2σ(*I*)
Graphite monochromator	*R*_int_ = 0.049
Detector resolution: 6.67 pixels mm^-1^	θ_max_ = 29.2°, θ_min_ = 2.8°
rotation method scans	*h* = −16→15
Absorption correction: for a sphere [modification of the interpolation procedure of Dwiggins (1975)]	*k* = −9→11
*T*_min_ = 0.090, *T*_max_ = 0.117	*l* = −19→19
9876 measured reflections	

### Refinement

**Table:**

Refinement on *F*^2^	Primary atom site location: structure-invariant direct methods
Least-squares matrix: full	Secondary atom site location: difference Fourier map
*R*[*F*^2^ > 2σ(*F*^2^)] = 0.038	Hydrogen site location: inferred from neighbouring sites
*wR*(*F*^2^) = 0.088	H atoms treated by a mixture of independent and constrained refinement
*S* = 1.11	*w* = 1/[σ^2^(*F*_o_^2^) + (0.0461*P*)^2^ + 0.1469*P*] where *P* = (*F*_o_^2^ + 2*F*_c_^2^)/3
3848 reflections	(Δ/σ)_max_ = 0.001
186 parameters	Δρ_max_ = 0.41 e Å^−^^3^
0 restraints	Δρ_min_ = −0.83 e Å^−^^3^

### Special details

**Table:**

Experimental. Absorption correction: interpolation using International Tables Vol C, Table 6.3.3.3 for values of muR in the range 0-2.5, and International Tables Vol. II, Table 5.3.6 B for µR in the range 2.6-10.0. The interpolation procedure (Dwiggins, 1975) is used with some modification.
Geometry. All e.s.d.'s (except the e.s.d. in the dihedral angle between two l.s. planes) are estimated using the full covariance matrix. The cell e.s.d.'s are taken into account individually in the estimation of e.s.d.'s in distances, angles and torsion angles; correlations between e.s.d.'s in cell parameters are only used when they are defined by crystal symmetry. An approximate (isotropic) treatment of cell e.s.d.'s is used for estimating e.s.d.'s involving l.s. planes.
Refinement. Refinement of *F*^2^ against ALL reflections. The weighted *R*-factor *wR* and goodness of fit *S* are based on *F*^2^, conventional *R*-factors *R* are based on *F*, with *F* set to zero for negative *F*^2^. The threshold expression of *F*^2^ > σ(*F*^2^) is used only for calculating *R*-factors(gt) *etc*. and is not relevant to the choice of reflections for refinement. *R*-factors based on *F*^2^ are statistically about twice as large as those based on *F*, and *R*- factors based on ALL data will be even larger.

### Fractional atomic coordinates and isotropic or equivalent isotropic displacement parameters (Å^2^)

**Table:**

	*x*	*y*	*z*	*U*_iso_*/*U*_eq_	
Br	0.515026 (19)	0.18718 (4)	0.289067 (16)	0.03329 (9)	
S	1.07362 (5)	0.49527 (7)	0.18671 (4)	0.02323 (12)	
N1	0.97746 (14)	0.3026 (2)	0.07003 (11)	0.0188 (3)	
N2	1.16473 (15)	0.3560 (2)	0.03586 (13)	0.0234 (4)	
O1	0.81067 (15)	0.2233 (2)	−0.04145 (11)	0.0267 (3)	
H1	0.878 (3)	0.246 (5)	−0.020 (2)	0.046 (10)*	
O2	1.36387 (13)	0.2471 (2)	−0.04068 (13)	0.0293 (4)	
C1	0.78829 (17)	0.2697 (2)	0.12458 (13)	0.0177 (4)	
C2	0.74833 (18)	0.2161 (2)	0.03574 (14)	0.0197 (4)	
C3	0.6411 (2)	0.1562 (3)	0.02405 (15)	0.0243 (4)	
H3	0.6152	0.1216	−0.0362	0.029*	
C4	0.57136 (19)	0.1461 (3)	0.09870 (16)	0.0246 (4)	
H4	0.4977	0.1064	0.0901	0.030*	
C5	0.61071 (17)	0.1947 (3)	0.18592 (15)	0.0228 (4)	
C6	0.71723 (17)	0.2532 (3)	0.20010 (14)	0.0207 (4)	
H6	0.7428	0.2826	0.2614	0.025*	
C7	0.89932 (17)	0.3417 (2)	0.13651 (13)	0.0175 (4)	
C8	0.93562 (18)	0.4462 (3)	0.20449 (13)	0.0202 (4)	
C9	0.8786 (2)	0.5237 (3)	0.28400 (15)	0.0287 (5)	
H9A	0.7991	0.5347	0.2680	0.043*	
H9B	0.9109	0.6299	0.2959	0.043*	
H9C	0.8885	0.4574	0.3403	0.043*	
C10	1.07224 (17)	0.3740 (3)	0.08740 (13)	0.0192 (4)	
C11	1.16493 (19)	0.2173 (3)	−0.02754 (16)	0.0264 (5)	
H11A	1.0941	0.2138	−0.0650	0.032*	
H11B	1.1712	0.1173	0.0096	0.032*	
C12	1.2614 (2)	0.2291 (3)	−0.09224 (16)	0.0306 (5)	
H12A	1.2643	0.1312	−0.1313	0.037*	
H12B	1.2501	0.3221	−0.1346	0.037*	
C13	1.3625 (2)	0.3931 (3)	0.01061 (19)	0.0319 (5)	
H13A	1.3499	0.4837	−0.0333	0.038*	
H13B	1.4356	0.4091	0.0433	0.038*	
C14	1.27235 (18)	0.3918 (3)	0.08152 (17)	0.0277 (5)	
H14A	1.2898	0.3100	0.1300	0.033*	
H14B	1.2691	0.4976	0.1127	0.033*	

### Atomic displacement parameters (Å^2^)

**Table:**

	*U*^11^	*U*^22^	*U*^33^	*U*^12^	*U*^13^	*U*^23^
Br	0.01940 (12)	0.04914 (17)	0.03197 (13)	−0.00366 (10)	0.01020 (8)	0.00066 (10)
S	0.0216 (2)	0.0252 (3)	0.0229 (2)	−0.0066 (2)	0.00275 (18)	−0.00190 (19)
N1	0.0156 (8)	0.0218 (8)	0.0193 (7)	0.0014 (7)	0.0033 (6)	0.0000 (6)
N2	0.0166 (8)	0.0258 (9)	0.0283 (8)	0.0011 (7)	0.0060 (7)	0.0003 (7)
O1	0.0285 (9)	0.0336 (9)	0.0184 (6)	−0.0062 (7)	0.0059 (6)	−0.0048 (6)
O2	0.0178 (7)	0.0303 (9)	0.0402 (9)	0.0059 (7)	0.0060 (7)	0.0021 (7)
C1	0.0160 (9)	0.0180 (9)	0.0192 (8)	0.0005 (7)	0.0012 (7)	−0.0009 (7)
C2	0.0229 (10)	0.0171 (9)	0.0192 (8)	−0.0003 (8)	0.0026 (7)	−0.0006 (7)
C3	0.0262 (11)	0.0233 (11)	0.0230 (9)	−0.0043 (8)	−0.0024 (8)	−0.0023 (8)
C4	0.0185 (10)	0.0227 (11)	0.0325 (10)	−0.0032 (8)	−0.0008 (8)	0.0027 (8)
C5	0.0166 (9)	0.0269 (10)	0.0251 (9)	−0.0001 (8)	0.0052 (7)	0.0003 (8)
C6	0.0172 (9)	0.0255 (10)	0.0196 (8)	0.0008 (8)	0.0025 (7)	−0.0016 (7)
C7	0.0178 (9)	0.0186 (9)	0.0162 (8)	0.0013 (7)	0.0021 (7)	0.0016 (7)
C8	0.0203 (9)	0.0220 (9)	0.0184 (8)	−0.0039 (8)	0.0030 (7)	0.0006 (7)
C9	0.0338 (12)	0.0316 (12)	0.0211 (9)	−0.0071 (10)	0.0091 (8)	−0.0055 (8)
C10	0.0185 (9)	0.0198 (9)	0.0193 (8)	0.0019 (8)	0.0020 (7)	0.0027 (7)
C11	0.0210 (10)	0.0330 (12)	0.0257 (10)	0.0009 (9)	0.0062 (8)	−0.0036 (9)
C12	0.0223 (11)	0.0424 (14)	0.0275 (10)	0.0093 (10)	0.0076 (9)	0.0050 (9)
C13	0.0199 (10)	0.0258 (12)	0.0506 (14)	0.0006 (9)	0.0108 (10)	0.0054 (10)
C14	0.0169 (10)	0.0257 (11)	0.0407 (12)	−0.0002 (8)	0.0047 (9)	−0.0017 (9)

### Geometric parameters (Å, º)

**Table:**

Br—C5	1.901 (2)	C4—H4	0.9500
S—C8	1.736 (2)	C5—C6	1.379 (3)
S—C10	1.742 (2)	C6—H6	0.9500
N1—C10	1.302 (3)	C7—C8	1.365 (3)
N1—C7	1.397 (2)	C8—C9	1.493 (3)
N2—C10	1.363 (3)	C9—H9A	0.9800
N2—C14	1.460 (3)	C9—H9B	0.9800
N2—C11	1.470 (3)	C9—H9C	0.9800
O1—C2	1.356 (2)	C11—C12	1.511 (3)
O1—H1	0.88 (4)	C11—H11A	0.9900
O2—C12	1.421 (3)	C11—H11B	0.9900
O2—C13	1.422 (3)	C12—H12A	0.9900
C1—C6	1.406 (3)	C12—H12B	0.9900
C1—C2	1.414 (3)	C13—C14	1.509 (3)
C1—C7	1.469 (3)	C13—H13A	0.9900
C2—C3	1.388 (3)	C13—H13B	0.9900
C3—C4	1.381 (3)	C14—H14A	0.9900
C3—H3	0.9500	C14—H14B	0.9900
C4—C5	1.378 (3)		
			
C8—S—C10	89.99 (10)	C8—C9—H9B	109.5
C10—N1—C7	111.64 (17)	H9A—C9—H9B	109.5
C10—N2—C14	117.64 (18)	C8—C9—H9C	109.5
C10—N2—C11	115.96 (18)	H9A—C9—H9C	109.5
C14—N2—C11	114.56 (18)	H9B—C9—H9C	109.5
C2—O1—H1	105 (2)	N1—C10—N2	124.91 (19)
C12—O2—C13	109.38 (18)	N1—C10—S	113.85 (14)
C6—C1—C2	117.42 (19)	N2—C10—S	121.22 (17)
C6—C1—C7	121.71 (18)	N2—C11—C12	110.0 (2)
C2—C1—C7	120.87 (17)	N2—C11—H11A	109.7
O1—C2—C3	117.13 (19)	C12—C11—H11A	109.7
O1—C2—C1	122.35 (19)	N2—C11—H11B	109.7
C3—C2—C1	120.50 (18)	C12—C11—H11B	109.7
C4—C3—C2	121.0 (2)	H11A—C11—H11B	108.2
C4—C3—H3	119.5	O2—C12—C11	111.13 (19)
C2—C3—H3	119.5	O2—C12—H12A	109.4
C5—C4—C3	118.7 (2)	C11—C12—H12A	109.4
C5—C4—H4	120.6	O2—C12—H12B	109.4
C3—C4—H4	120.6	C11—C12—H12B	109.4
C4—C5—C6	121.68 (19)	H12A—C12—H12B	108.0
C4—C5—Br	119.49 (16)	O2—C13—C14	111.12 (19)
C6—C5—Br	118.81 (16)	O2—C13—H13A	109.4
C5—C6—C1	120.55 (19)	C14—C13—H13A	109.4
C5—C6—H6	119.7	O2—C13—H13B	109.4
C1—C6—H6	119.7	C14—C13—H13B	109.4
C8—C7—N1	115.22 (18)	H13A—C13—H13B	108.0
C8—C7—C1	127.65 (18)	N2—C14—C13	110.4 (2)
N1—C7—C1	117.12 (17)	N2—C14—H14A	109.6
C7—C8—C9	132.3 (2)	C13—C14—H14A	109.6
C7—C8—S	109.29 (15)	N2—C14—H14B	109.6
C9—C8—S	118.36 (16)	C13—C14—H14B	109.6
C8—C9—H9A	109.5	H14A—C14—H14B	108.1
			
C6—C1—C2—O1	−178.6 (2)	N1—C7—C8—S	−1.2 (2)
C7—C1—C2—O1	2.2 (3)	C1—C7—C8—S	−179.83 (17)
C6—C1—C2—C3	2.7 (3)	C10—S—C8—C7	0.98 (16)
C7—C1—C2—C3	−176.49 (19)	C10—S—C8—C9	−177.13 (18)
O1—C2—C3—C4	−179.3 (2)	C7—N1—C10—N2	178.48 (19)
C1—C2—C3—C4	−0.6 (3)	C7—N1—C10—S	−0.1 (2)
C2—C3—C4—C5	−0.9 (3)	C14—N2—C10—N1	−157.2 (2)
C3—C4—C5—C6	0.2 (4)	C11—N2—C10—N1	−16.2 (3)
C3—C4—C5—Br	178.34 (17)	C14—N2—C10—S	21.2 (3)
C4—C5—C6—C1	2.0 (4)	C11—N2—C10—S	162.21 (16)
Br—C5—C6—C1	−176.16 (17)	C8—S—C10—N1	−0.55 (17)
C2—C1—C6—C5	−3.4 (3)	C8—S—C10—N2	−179.14 (19)
C7—C1—C6—C5	175.8 (2)	C10—N2—C11—C12	169.71 (19)
C10—N1—C7—C8	0.9 (3)	C14—N2—C11—C12	−48.1 (3)
C10—N1—C7—C1	179.61 (18)	C13—O2—C12—C11	−62.9 (3)
C6—C1—C7—C8	−23.9 (3)	N2—C11—C12—O2	54.7 (3)
C2—C1—C7—C8	155.3 (2)	C12—O2—C13—C14	62.7 (3)
C6—C1—C7—N1	157.5 (2)	C10—N2—C14—C13	−170.3 (2)
C2—C1—C7—N1	−23.3 (3)	C11—N2—C14—C13	48.2 (3)
N1—C7—C8—C9	176.5 (2)	O2—C13—C14—N2	−54.7 (3)
C1—C7—C8—C9	−2.1 (4)		

### Hydrogen-bond geometry (Å, º)

**Table:**

*D*—H···*A*	*D*—H	H···*A*	*D*···*A*	*D*—H···*A*
O1—H1···N1	0.88 (4)	1.79 (3)	2.603 (2)	154 (3)
C9—H9*C*···O1^i^	0.98	2.47	3.357 (3)	150

Symmetry code: (i) *x*, −*y*+1/2, *z*+1/2.
